# Attention Orienting in Response to Non-conscious Hierarchical Arrows: Individuals with Higher Autistic Traits Differ in Their Global/Local Bias

**DOI:** 10.3389/fpsyg.2017.00023

**Published:** 2017-01-18

**Authors:** Robin Laycock, Daniel Chan, Sheila G. Crewther

**Affiliations:** School of Psychology and Public Health, La Trobe University, MelbourneVIC, Australia

**Keywords:** autism spectrum disorder, attention, non-conscious processing, continuous flash suppression, local/global processing

## Abstract

One aspect of the social communication impairments that characterize autism spectrum disorder (ASD) include reduced use of often subtle non-verbal social cues. People with ASD, and those with self-reported sub-threshold autistic traits, also show impairments in rapid visual processing of stimuli unrelated to social or emotional properties. Hence, this study sought to investigate whether perceptually non-conscious visual processing is related to autistic traits. A neurotypical sample of thirty young adults completed the Subthreshold Autism Trait Questionnaire and a Posner-like attention cueing task. Continuous Flash Suppression (CFS) was employed to render incongruous hierarchical arrow cues perceptually invisible prior to consciously presented targets. This was achieved via a 10 Hz masking stimulus presented to the dominant eye that suppressed information presented to the non-dominant eye. Non-conscious arrows consisted of local arrow elements pointing in one direction, and forming a global arrow shape pointing in the opposite direction. On each trial, the cue provided either a valid or invalid cue for the spatial location of the subsequent target, depending on which level (global or local) received privileged attention. A significant autism-trait group by global cue validity interaction indicated a difference in the extent of non-conscious local/global cueing between groups. Simple effect analyses revealed that whilst participants with lower autistic traits showed a global arrow cueing effect, those with higher autistic traits demonstrated a small local arrow cueing effect. These results suggest that non-conscious processing biases in local/global attention may be related to individual differences in autistic traits.

## Introduction

Autism spectrum disorder (ASD) is defined by DSM-5 as representative of persistent deficits in social communication and interaction, including deficits in social-emotional reciprocity, non-verbal communication, and repetitive patterns of behavior. Social and emotional processing impairments have long been associated with a deficiency in brain activation of subcortical emotion networks (e.g., Baron-Cohen et al., 2000; Hall et al., 2007; Kleinhans et al., 2011; Hernandez et al., 2015), though there is now also strong evidence for visual perceptual abnormalities of many stimuli types across the autism spectrum (for reviews, see Dakin and Frith, 2005; Laycock et al., 2007; Simmons et al., 2009; Crewther et al., 2015).

Skilled social interactions rely on the detection and interpretation of changes in non-verbal cues. Many of these social signals may be processed implicitly, without direct conscious awareness and are reported to be impaired in ASD (e.g., Senju et al., 2008; Schwartz et al., 2010; Schilbach et al., 2012). For example, electrophysiology and brain imaging studies have found that ASD groups show abnormal neural responses to implicit or non-conscious emotion processing when contrasting emotional and neutral faces (i.e., explicit attention is directed to non-emotional aspects of the stimuli, such as being required to make a gender discrimination) (e.g., Batty et al., 2011; Spencer et al., 2011; Nuske et al., 2014). Others have emphasized impairments in both explicit and implicit emotion processing (e.g., Critchley et al., 2000; Wong et al., 2008; Kuchinke et al., 2011).

The interaction between conscious and non-conscious visual processing of social information in ASD adolescents has recently been examined by making use of Continuous Flash Suppression (CFS) (Akechi et al., 2014). CFS is an interocular suppression technique that facilitates lasting suppression of visual stimuli from conscious awareness (Tsuchiya and Koch, 2005). In their study, Akechi et al. (2014) recorded the time for suppressed faces with either a direct or an averted gaze to reach conscious awareness (i.e., break suppression). Direct gaze faces reached conscious awareness faster than averted gaze faces in control participants, however, ASD adolescents did not detect direct gaze faces faster than averted gaze faces. Furthermore, ASD participants did not differ from controls in a conscious detection task, with both groups demonstrating a direct gaze advantage. Thus abnormal performance in ASD participants was only demonstrated in the non-conscious tasks, indicating weaker, initial non-conscious registration of eye contact.

Despite the ASD literature commonly describing anomalies in perceptual and cognitive processing of affective stimuli (e.g., Harms et al., 2010; Uljarevic and Hamilton, 2013; Lozier et al., 2014), there is also strong evidence for basic-level, non-affective perceptual processing abnormalities in motion perception, contrast sensitivity, and global processing for individuals on the autism spectrum (e.g., Spencer et al., 2000; Pellicano et al., 2005; Grinter et al., 2009; Greenaway and Plaisted-Grant, 2013), although these visual impairments have not always been replicated (e.g., Milne et al., 2006; Saygin et al., 2010; Jones et al., 2011). Anomalous visual processing in ASD also appears to include superior performance on tasks requiring attention to small details or local-level processing such as visual search and the embedded figures test (e.g., Bertone et al., 2005; Grinter et al., 2009). This local/global abnormality has become the focus of much research in ASD (e.g., Robertson et al., 2013; Ronconi et al., 2013).

A further area of non-social processing in ASD receiving research interest is that of selective attention. Mixed findings have made reaching a consensus difficult. For example, although two more recent findings have suggested that ASD adults show no differences in spatial attention (Grubb et al., 2013a,b), a slightly earlier meta-analysis by Landry and Parker (2013) concluded that ASD was associated with a reduced magnitude of the cueing effect in Posner-type attention tasks (Posner, 1980) compared to controls. This effect was reportedly most pronounced when utilizing arrow cue tasks.

Thus, given the apparent deficiency in affective non-conscious processing in ASD, there remains a need for better understanding of whether this deficiency might also be established for non-affective stimuli. In particular, the question of whether differences in local/global visual processing, as well as attention orienting, are apparent without conscious awareness, can contribute to an understanding of the range of non-conscious processing abnormalities on the autism spectrum.

There is now a growing literature demonstrating similar visual and cognitive anomalies in what is sometimes termed the broader autism phenotype (Piven et al., 1997) to that found in individuals with an ASD diagnosis (e.g., Dalton et al., 2007; Grinter et al., 2009; Almeida et al., 2014). For example, it has previously been shown that unaffected siblings of individuals with an ASD show similar atypical eye-fixation patterns to faces as well as similar reductions in amygdala volume (Dalton et al., 2007). In particular, there is evidence for a similar pattern of anomalous local/global visual processing in populations with sub-clinical range autism traits (e.g., Grinter et al., 2009; Almeida et al., 2010; Crewther and Crewther, 2014; Crewther et al., 2015) suggesting a continuum between clinically diagnosed ASD and typically developing individuals with higher autism-like characteristics. These findings also suggest that similar neural mechanisms might be underpinning the perceptual and cognitive styles in clinical and subthreshold ASD populations.

**FIGURE 1:**
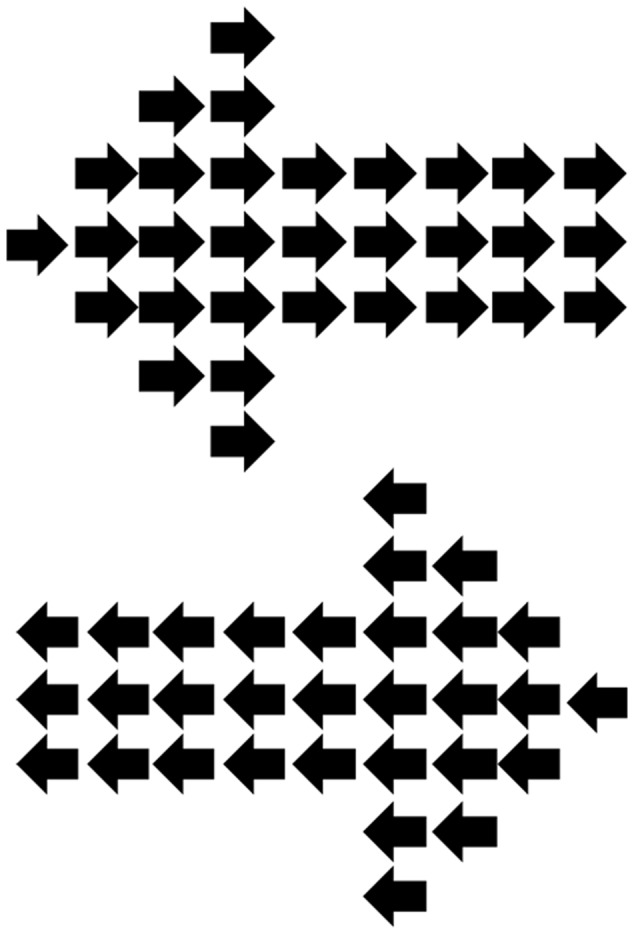
**Hierarchical local/global arrow stimuli used during the continuous flash suppression (CFS) display.** The global-level arrow was always incongruent with the local-level arrows.

Here, we used an adaptation of Navon’s more commonly used local/global hierarchical stimuli (Navon, 1983), involving a global arrow composed of smaller local arrows (see **Figure 1** in Methods). We utilized these local/global arrows in a traditional spatial attention cueing paradigm (Posner, 1980). The paradigm involves a spatial cue providing information about the location of a subsequent target appearing in the periphery. Targets appear either at the cued (valid condition) or at a different (invalid condition) location. Although many studies have used either exogenous or endogenous cues, more recent studies suggest that arrow cues are a category of their own, providing an automatic facilitation of attention to the cued location (Ristic et al., 2002; Tipples, 2002; Galfano et al., 2012). Importantly this advantage of attention orienting to the cued compared with the non-cued location occurs even when the arrow direction is non-predictive (i.e., 50% of trials validly cued) (Tipples, 2002).

Mills and Dodd (2014) demonstrated that healthy adults revealed a global precedence effect when spatial attention was cued by local/global arrows at shorter onset latencies but a local bias in attentional cueing with a longer onset delay between cue and target. An advantage of utilizing these hierarchical stimuli in such a cueing task is that the arrow cues can be non-predictive of subsequent target location, and furthermore any given cue consists of both valid and invalid information of target location depending on whether local or global information is prioritized. Thus the extent to which a natural disposition toward utilizing global or local information can be determined (Mills and Dodd, 2014). We sought to investigate the extent to which non-conscious cueing by local/global arrows might show a relationship with individual differences in sub-threshold autistic traits.

The current study makes an assumption that, regardless of autistic traits, non-conscious cueing of spatial attention is in fact achievable. This perhaps unexpected possibility rests on the contention that attention and consciousness are separable processes that might even be viewed as orthogonal (Lamme, 2003; Koch and Tsuchiya, 2007). For example, perceptually unseen low luminance cues can still capture attention automatically in healthy observers (McCormick, 1997). Kentridge et al. (1999) also explored non-conscious visual attention in a blindsight patient and demonstrated that exogenous cues presented in the blind visual field were capable of directing attention.

To our knowledge, only one previous study has explored aspects of non-conscious processing in a healthy population varying in autistic traits (Hudson et al., 2012). In that study implicit learning of visible pro-social or anti-social expressive face identities were subsequently shown to differentially influence responses in a gaze-cueing task between high- and low-autism trait groups. In the current study, we sought to more directly explore processing of stimuli not consciously perceived. By using incongruous hierarchical arrow cues that were suppressed from awareness, the cues could thus be simultaneously valid at the global level, but invalid at the local level (and vice versa). The extent to which valid compared with invalid global/local conditions conferred a reaction time (RT) advantage could be assumed to be a function of the non-conscious focus of attention. We expected that those individuals with low autistic traits would demonstrate a global bias, and conversely that high autistic-trait participants would demonstrate a local bias in non-conscious attentional cueing.

## Materials and Methods

### Participants

Thirty-seven healthy young adults (mean age = 24.78, *SD* = 5.12) with no known diagnosis of ASD or other neurological or psychiatric condition participated in the study (24 females, 13 males). The study had approval from the Human Ethics Committee of the Faculty of Science Technology and Engineering at La Trobe University, with all methods carried out in accordance with the approved guidelines. All participants provided written informed consent in accordance with the Declaration of Helsinki.

Participants had their self-reported autism traits assessed by completing the Subthreshold Autism Trait Questionnaire (SATQ) (Kanne et al., 2012). The SATQ is a 24-item questionnaire that utilizes the fact that individuals will differ in their social and communication skills, and in fact in the broader population autistic traits are continuously distributed with no discrete separation between a clinical diagnosis and the sub-clinical population (Kanne et al., 2012; Nishiyama et al., 2014). The SATQ is argued to be suitable for use in the general population and assesses a broad range of ASD-related symptoms (Kanne et al., 2012). A recent comparative study of a large sample (*n* = 3,147) showed that the SATQ had good internal reliability (Cronbach’s alpha = 0.79) and test–retest reliability (0.87) comparing favorably with other common self-report autism phenotype questionnaires (Nishiyama et al., 2014). Nishiyama et al. (2014) concluded that some questionnaires examined did not have strong discriminative properties and the use of the SATQ was recommended.

### Stimuli

The experiment, which was designed using VPixx Technologies^1^ took the form of a Posner-type cueing experiment (Posner, 1980), involving an arrow cue followed by a simple target presented to the left or right. In the current study, however, cue stimuli were suppressed from conscious awareness by the use of CFS. Cues were hierarchical local/global arrows, following the commonly used hierarchical stimuli consisting of a larger (global) item formed out of smaller (local) items (Navon, 1983). Usually these Navon stimuli involve the global and local levels being either congruent or incongruent, however, in the current experiment the global and local arrows were always incongruent (e.g., global left, local right, see **Figure 1**). These arrow stimuli, similar to that used previously (Weinbach and Henik, 2011), thus consisted of a number of smaller arrows (each subtending 0.6° length, 0.25° height at body, 0.5° height at widest part of arrow head) forming the global shape of a larger arrow (5.3° length, 1.5° height at the arrow body, 4° height at the widest part of the arrow head).

**FIGURE 2:**
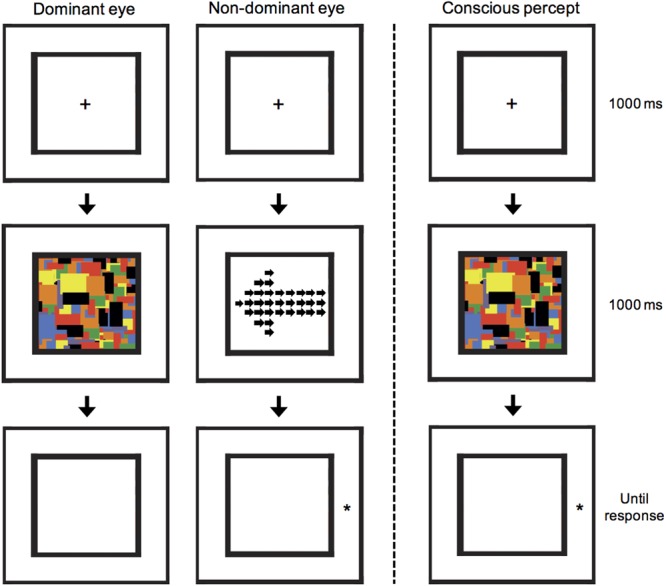
**A schematic illustration of the CFS arrow-cuing task.** A local/global arrow was presented to the non-dominant eye, but suppressed from awareness by presenting a 10 Hz flashing series of Mondrian images to the dominant eye. Subsequently, participants were shown a target stimulus and asked to press a button as quickly as possible to indicate whether it appeared on the left or right. In the illustrated example, a global invalid condition is shown, in which the target appears in the opposite location to that cued by the global-level arrow cue. This example can also be described as a local valid condition, in which the target appears in the same location to that cued by the local-level arrows.

In order to generate the CFS effect, we used a set of 10 colored Mondrian images presented on a mid-gray background, and enclosed in a black frame which subtended 5.9° × 5.9° of visual angle. Mondrian patterns are composed of an irregular array of multi-colored squares and rectangles of different sizes and orientations (see **Figure 2**). A small red circle (0.2° diameter) was displayed in front of each Mondrian as a central fixation point. The arrow cueing stimulus was also displayed inside an identical black frame in order that the fusion of the two retinal displays would be facilitated. The target stimulus was a star symbol (^∗^) written in 30-point font, and displaced 4° left or right from the center of the black frame (i.e., approximately 1° outside the frame).

### Procedure

For all CFS stimuli, the Mondrian masking stimuli were presented to the participants’ dominant eye at a rate of 10 Hz (Tsuchiya and Koch, 2005), at the same time as cue stimuli were presented to the non-dominant eye. Eye dominance was first established by use of the Porta test in which participants extend one arm and align thumb and finger with a mark on the wall with both eyes open. The participant then alternates closing each eye to determine which eye is viewing the object (i.e., the dominant eye). Before the cueing experiment, each participant completed a pre-experimental control to firstly determine the highest contrast of the arrow stimulus that would still result in reliable suppression. If luminance contrast of the cue is too high, then stimuli break through the Mondrian masks and suppression is not achieved. In this experiment, an arrow was presented in either the left or right half of the black frame; the arrow stimulus dimensions were modified to fill a 3.3° × 3.3° space in only half of the frame. The arrow stimulus had its contrast linearly ramped on over a 1000 ms duration after which the Mondrian images ceased and a white noise mask was displayed.

In the control experiment, text on the screen prompted the participant to indicate manually whether the arrow stimulus had been presented to the left or right of the center of the frame. Thus a detection task was used to be more conservative than requiring participants to discriminate the direction of the arrow. After responding, participants were asked a second question, “did you see anything at all? Yes or no.” Before the experiment it was explained to participants that sometimes they might not be sure what side the arrow was on, but they might nevertheless have seen even a small portion of the arrow (partial breakthrough), in which case they should respond “yes.” Responses were made with a button press using both index fingers on a RESPONSEPixx button box^2^. The button box format consists of a diamond shape of buttons (left/right, up/down). On each trial the instructions reminded participants that the up button indicated “yes” and the down button indicated “no.”

A method of constant stimuli approach was used with 10 trials presented (five left, five right) at three different contrasts for a total of 30 trials. The aim of this testing was to establish the highest possible luminance contrast at which suppression from awareness was still achieved. Luminance contrast was defined in VPixx software as the contrast percentage difference between the peak and trough of the stimulus around a mid-gray RGB saturation. If after the first block of 30 trials participants were at chance level (i.e., 50% correct) then a second block of trials was repeated with three higher contrast levels selected. If on the other hand performance was above chance for all contrast levels tested, the test was repeated with three lower contrast levels selected. Once the highest contrast level that produced chance performance was determined, a further 40 trials were completed (20 left, 20 right) at the selected contrast serving as the pre-experimental control test. Across all participants the mean luminance contrast was 10.9% (*SD* = 4.2). However, the luminance contrast level used for each participant during the experiment was individually determined.

After completing the pre-experimental control, the cueing experiment was explained to participants, and a practice block of 40 trials was completed to ensure the participant understood the task and CFS was functioning as intended. The cueing experiment consisted of 50% valid trials in which the global level of the arrow correctly cued the side at which the target star would appear, and 50% invalid trials in which the global level cued the incorrect target location. Both cue direction (left or right pointing) and target location (left or right side) occurred with equal probability. Note that whilst the global-level arrow may be a valid cue, at the local-level the same stimulus is an invalid cue, and vice versa. Participants were not given information about whether the arrows would be predictive, as these stimuli were suppressed from awareness. After the 1000 ms 10 Hz Mondrians, instead of a white-noise mask, the final Mondrian presented to the dominant eye was removed and instead presented to the non-dominant eye as a mask. This was done to prevent the arrow stimulus leaving an afterimage that was sometimes reported in piloting. Thus, during the task, participants consciously perceived an empty black frame, followed by flashing Mondrians for 1 s, after which a star appeared to the side of the final Mondrian image (see **Figure 2**). Participants were asked to respond as quickly and accurately as possible to the side with which the star appeared using a button press. Similar to the presentation in the control experiment, the arrow cue stimulus was ramped on over the full 1000 ms reaching the individual maximum contrast. Two blocks of 40 trials were completed and analyzed. Within a block all four conditions were fully randomized. Finally, after completing the cueing experiment, the control experiment was re-run with exactly the same design as described for the pre-experimental control, using the same contrast as used during the cueing experiment in order to confirm that suppression was maintained.

Pre- and post-control testing revealed that the proportion of correct responses for identifying the stimulus location, or the proportion of trials with arrow visibility reported, was deemed to be too high in seven participants and thus indicated unreliable suppression. For these participants, the proportion of correct responses that were expected to be at chance assuming complete suppression averaged 73% (range: 60–97%) for the pre-control test, and 61% (range: 42–75%) for the post-control test. The proportion of trials with an arrow reported as visible averaged 44% (range: 3-82%) for the pre-control test, and 49% (range: 35–60%) for the post-control test. These participants were excluded from the following control experiment analyses as well as the main cueing experiment results.

## Results

### Pre- and Post-experimental Control Tests

The remaining 30 participants included in the analyses performed at a chance level in detecting which side of the screen an arrow stimulus appeared (pre-control test: *M* = 51.0%, *SD* = 8.9; post-control test: *M* = 52.5%, *SD* = 7.7). In addition, participants reported some part of the stimulus to be visible on very few trials (pre-control test: *M* = 3.8%, *SD* = 4.4; post-control test: *M* = 2.9%, *SD* = 4.0). These results indicate strong and reliable suppression.

### Non-conscious Arrow Cueing

During the main CFS cuing experiment, error rates in detecting the visible target were very small, with a mean accuracy of 99.29% (*SD* = 1.1). Hence, no further analysis of accuracy was made, with the focus on RT to respond to the target star on accurate trials.

The cueing effect was calculated by first collapsing data across left and right sided stimuli, and then subtracting mean RT for global-valid trials from that for global-invalid trials. This cueing effect was then averaged across both blocks of trials completed by each participant. In this way, a positive cueing effect would indicate a global cueing effect, whereas a negative cueing effect would indicate a local cueing effect. For example, a global-valid RT of 330 ms, and a global-invalid RT of 340 ms, produces a cueing effect of 10 ms indicating a global bias in orienting. Whereas a global-valid RT of 345 ms, and a global-invalid RT of 335 ms, produces a cueing effect of -10 ms. To clarify, given that all arrow cues were incongruous, this latter example could also be described as a local-invalid RT of 345 ms, and a local-valid RT of 335 ms, and hence local valid RT subtracted from local invalid RT produces a *local* cueing effect of 10 ms.

A correlation between SATQ score and the global cueing effect was explored to determine if higher autistic traits would predict a tendency away from global cuing toward local cueing. As predicted, a significant negative correlation was established, *r* = -0.392, *p* = 0.032. In addition, correlations were conducted between the cueing effect and the five SATQ factors established by Kanne et al. (2012). This showed that the global cueing effect correlated negatively with the Social Interaction and Enjoyment (*r* = -0.420, *p* = 0.021), and the Rigidity (*r* = -0.381, *p* = 0.038) subscales, but not with the Oddness, Reading Facial Expressions, and Expressive Language subscales.

To establish whether a significant global arrow cueing effect or a local arrow cueing effect was evident in low and high autistic-trait participants, respectively, a mixed design ANOVA was conducted with global cue validity (valid, invalid) as a within subject factor, and autism-trait group (low, high) as a between group factor. Low and high autism-trait groups were created by a median split (Med SATQ score = 22.5, see details in **Table 1**).

**Table 1:** Autism-trait group demographics.

	SATQ score (*SD*)	*N*	Gender ratio (M:F)
Low autism-trait Group	14.4 (5.2)	15	5:10
High autism-trait Group	29.13 (3.9)	15	8:7
Total sample	21.77 (8.8)	30	13:17

As more males than females are diagnosed clinically with ASD (e.g., Russell et al., 2011) the effect of gender on autistic traits was considered potentially important. A chi-squared test showed that autistic-trait group membership and gender were independent, χ^2^(1, *N* = 30) = 1.22, *p* = 0.269. However, given that our entire sample had more females than males (see **Table 1**) gender was nevertheless also included as a second between subject factor in the ANOVA.

ANOVA results showed that there was a main effect of gender, *F*(1,26) = 5.70, *p* = 0.024, $\eta_{p}^{2}$ = 0.180, with males showing slower mean RTs (*M* = 322 ms, *SD* = 15) than females (*M* = 377 ms, *SD* = 17). Importantly, for understanding the influence of gender on non-conscious processing, the gender by cue validity interaction was not significant *F*(1,26) = 0.03, *p* = 0.876, $\eta_{p}^{2}$ = 0.001, and similarly the three-way interaction between gender, autistic-trait group, and cue validity was also not significant, *F*(1,26) = 2.12, *p* = 0.157, $\eta_{p}^{2}$ = 0.075.

**FIGURE 3:**
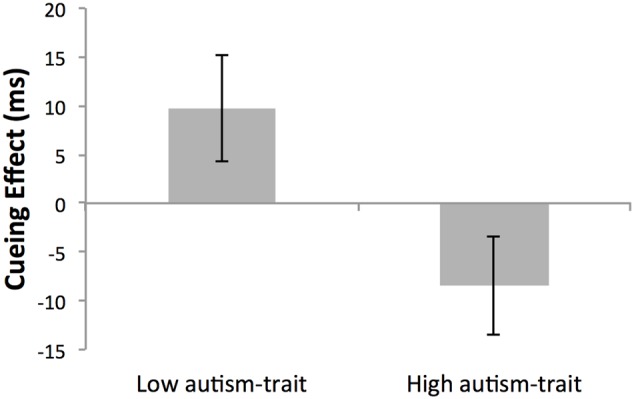
**Mean cueing effect (ms) ± SEM of non-conscious arrow cues in Low- and High- autism-trait groups.** The cueing effect was calculated as the mean reaction time for global validly cued trials subtracted from global invalidly cued trials. Given that all cue stimuli were incongruent at the global and local level, a positive cueing effect indicates a global bias, whilst a negative cueing effect indicates a local bias in non-conscious cueing.

Although there was no main effect of global cue validity (*p* = 0.866) or group (*p* = 0.305), the critical two-way interaction between cue validity and autistic-trait group was significant, *F*(1,26) = 5.93, *p* = 0.022, $\eta_{p}^{2}$ = 0.186 (see **Figure 3**). Simple main effects analyses revealed that as expected the low autistic-trait group demonstrated longer RTs for global-invalid (*M* = 343 ms, *SD* = 61) compared with global-valid (*M =* 333 ms, *SD* = 70) cueing conditions (*p* = 0.085, $\eta_{p}^{2}$ = 0.110), and had a mean global cueing effect of +9.70 ms. Conversely, the high autistic-trait group demonstrated shorter RTs for global-invalid (*M* = 358 ms, *SD* = 58) compared with global-valid cueing conditions (*M* = 365 ms, *SD* = 66) (*p* = 0.111, $\eta_{p}^{2}$ = 0.095), with a mean global cueing effect of -8.43 ms (recalling that a negative global cueing effect provides evidence for local arrow cueing). Although non-significant, the large and medium cueing effect sizes established for the low and high autistic-trait groups, respectively, does indicate a group difference in the relative bias toward local/global processing. This can be seen by the significant difference between autistic-trait groups in the absolute size of the global cueing effect using an independent samples *t*-test, *t*(28) = 2.28, *p* = 0.031, *d* = 0.83.

## Discussion

The aim of this study was to examine the relationship between non-conscious visual processing and autistic traits in a neurotypical non-clinical sample. It sought to determine whether anomalous biases in local/global processing previously found in consciously driven tasks on populations with higher autistic traits (e.g., Grinter et al., 2009; Crewther and Crewther, 2014), would persist without conscious awareness, perhaps reflective of a more generalized cognitive style. Results confirmed the hypothesis that lower autistic traits would be associated with a non-conscious global-level bias, and hence a cueing effect directed by the global level of a hierarchical arrow stimulus. Furthermore, as expected, participants with higher autistic traits showed a local bias in non-conscious processing. Thus these results provide evidence that the tendency toward local-level perceptual processing often reported for conscious visual processing in clinical and subthreshold ASD populations (e.g., Bertone et al., 2005; Grinter et al., 2009) may also apply to non-conscious processing.

As outlined in the Section “Introduction,” an underlying assumption of this study was that in healthy observers across all autism traits, non-conscious pathways are capable of facilitating spatial attention. Previous research utilizing stroke patients has suggested that non-conscious pathways may in fact be utilized for automatic attentional capture (Kentridge et al., 1999), though this is not the case for all cue types (Burra et al., 2014). In the current study, CFS was used to present hierarchical arrow cues non-consciously in a spatial cueing task. Notably, across the whole sample, 70% of participants showed a cueing effect greater than +8 ms, or less than -8 ms (i.e., either global or local cueing effects). This cueing effect compares favorably with the approximately 10–15 ms cueing effect reported in the nearly identical (though consciously presented) paradigm reported by Mills and Dodd (2014), and similarly with previous (conscious) arrow cueing studies of 10–20 ms (e.g., Ristic et al., 2002; Tipples, 2002; Galfano et al., 2012). The size of the cueing effects reported here are particularly impressive considering a smaller cueing effect might be expected from non-conscious compared with conscious arrow cueing.

Previous research suggests that individuals on the autism spectrum show either deficits in global processing, or enhancements in local processing (e.g., Bertone et al., 2005; Wang et al., 2007; Muth et al., 2014). To date this has not been specifically tested without conscious awareness. Although some caution in interpreting the current data is required, the significant interaction between group and global cue validity, and the opposing direction of the moderate-large sized effects for high and low autistic-trait groups (along with the significant correlation between SATQ scores and cueing effects), do support the contention that those with higher autistic traits show a greater tendency toward a local bias in non-conscious processing than do those with lower autistic traits. In this sense, the results are consistent with one of the most prominent models explaining the biases in ASD processing styles, referred to as the Weak Central Coherence model. Initially this model was conceptualized as a generalized cognitive approach in which deficits in global processing could produce local-level biases (Happe and Frith, 1996). The model continues to be debated (Mottron et al., 2006), with newer data tending to emphasize superior local, rather than inferior global, processing in ASD populations (Happe and Frith, 2006; Sutherland and Crewther, 2010).

The influence of spatial attention on local/global processing has also been considered. One suggestion is that enhanced perception of fine details in ASD drops off at a faster rate as stimuli move further from foveal vision, presenting as a form of “tunnel vision” in ASD (Robertson et al., 2013). Similarly, it has been shown that high and low autistic-trait groups did not differ in performance when viewing a bistable visual illusion, though high ASD-trait participants were less likely to report an initial global percept when the stimulus was presented further into the periphery (Crewther and Crewther, 2014).

Specific neural networks cannot be inferred from the current data. However, it is clear that individuals with higher autistic traits appear to process different aspects of salient information, including non-social cues, through a non-conscious visual network. This view implies that arrows, which in the real world often provide important information about danger or directions, constitute a salient goal-directed cue that is automatically processed.

Supporting the interpretation of non-emotional perceptual detection anomalies in ASD, we have recently demonstrated that a neurotypical population with higher autistic traits was relatively impaired in a visual object discrimination task (Laycock et al., 2014). Contrast threshold required for object discrimination was used to assess non-social object (e.g., chair, blender) processing in ‘abrupt’ and ‘ramped’ presentation conditions. Impaired performance, as indicated by higher contrast thresholds, by high compared with low autistic-trait participants was observed when object presentation was abrupt, but not when luminance contrast was gradually ramped on and off. The finding was interpreted as indicative of a possible impairment in the utilization of rapid attention mechanisms for sudden or salient environmental events. These deficiencies, if replicated in clinical samples, could indicate that both conscious and non-conscious pathways recruited to activate and direct visual attention may be impaired or at least function differently in autism. If conscious and non-conscious attentional systems are either slower to be initiated, or operate with a local attentional bias, then the impact of these factors on childhood development could well be expected to make interpreting a socially complex world more difficult. Consistent with this view, Keehn et al. (2013) have argued that deficits in disengaging attention can impact on the development of sociocommunicative functions, and thus ultimately the model proposes that attentional mechanisms in fact contribute to the emergence of the ASD symptoms.

As already noted, abnormal biases in local/global visual processing have been reported in individuals diagnosed with an ASD (e.g., Plaisted et al., 1999; Spencer et al., 2000; Pellicano et al., 2005; Bolte et al., 2007) and have also been established in neurotypical populations with higher autism-like characteristics (e.g., Grinter et al., 2009; Almeida et al., 2010; Crewther and Crewther, 2014; Crewther et al., 2015; Cribb et al., 2016). Nevertheless, although it is suggestive, as Gregory and Plaisted-Grant (2013) have argued, it cannot be assumed that the same underlying mechanism can explain a given perceptual abnormality in clinical and non-clinical groups. Moreover, caution should be exercised having revealed a previously unknown anomaly – in the current case non-conscious global/local cueing – in a neurotypical sample, before extrapolating to a clinical population. As such, the current finding will require replication in a clinical population. In addition, future work should endeavor to directly compare both affective and non-affective non-conscious processing in the same population of individuals varying in autistic traits.

To conclude, the current results demonstrate that non-predictive hierarchical arrow stimuli presented without awareness can promote spatial cueing effects, reinforcing the suggestion that arrows act as an automatic trigger for directing attention (Tipples, 2002). Importantly, this conclusion, and the extent to which cueing was driven by the global- or local-level arrows, is moderated by individual differences in sub-threshold autistic traits. Namely, the commonly described reduction in global processing and superiority in local processing previously found in ASD and sub-threshold high autistic-trait populations (e.g., Wang et al., 2007; Grinter et al., 2009) appears to also be a feature of non-conscious processing in the sub-threshold autistic-trait population tested here.

Previous research has proposed that abnormalities in non-conscious processing by those on the autism spectrum, including those with ASD might contribute to abnormal analysis of, or alerting to, subtle non-verbal social cues (e.g., Critchley et al., 2000; Hudson et al., 2012); we suggest that such non-conscious processing impairments of social or emotional attributes might reflect only an element of a more general difference in non-conscious processing in the autism spectrum. In fact, the evidence presented here indicates that a more locally biased cognitive approach in those with higher autistic traits occurs not just during consciously driven tasks, but potentially also without conscious awareness.

## Author Contributions

RL designed the experiments. RL and DC collected and analyzed the data. RL, DC, and SC interpreted the data. RL and SC wrote the main manuscript text. All authors reviewed the manuscript.

## Conflict of Interest Statement

The authors declare that the research was conducted in the absence of any commercial or financial relationships that could be construed as a potential conflict of interest.

## Footnotes

1 http://vpixx.com

2 http://vpixx.com
